# Retroductal dexamethasone administration promotes the recovery from obstructive and inflammatory salivary gland dysfunction

**DOI:** 10.3389/fimmu.2024.1418703

**Published:** 2024-07-09

**Authors:** Seungyeon Hwang, Jae-Min Cho, Yeo-Jun Yoon, Sunyoung Seo, Yongpyo Hong, Jae-Yol Lim

**Affiliations:** ^1^ Department of Otorhinolaryngology, Yonsei University College of Medicine, Seoul, Republic of Korea; ^2^ Gangnam Severance Hospital, Yonsei University College of Medicine, Seoul, Republic of Korea

**Keywords:** salivary gland, obstructive sialadenitis, corticosteroids, regeneration, stem cell, organoids

## Abstract

**Introduction:**

Salivary gland dysfunction, often resulting from salivary gland obstruction-induced inflammation, is a prevalent condition. Corticosteroid, known for its anti-inflammatory and immunomodulatory properties, is commonly prescribed in clinics. This study investigates the therapeutic implications and potential side effects of dexamethasone on obstructive sialadenitis recovery using duct ligation mice and salivary gland organoid models.

**Methods:**

Functional and pathological changes were assessed after administering dexamethasone to the duct following deligation 2 weeks after maintaining ligation of the mouse submandibular duct. Additionally, lipopolysaccharide- and tumor necrosis factor-induced salivary gland organoid inflammation models were established to investigate the effects and underlying mechanisms of action of dexamethasone.

**Results:**

Dexamethasone administration facilitated SG function restoration, by increasing salivary gland weight and saliva volume while reducing saliva lag time. Histological evaluation revealed, reduced acinar cell atrophy and fibrosis with dexamethasone treatment. Additionally, dexamethasone suppressed pro-inflammatory cytokines IL-1β and TNF expression. In a model of inflammation in salivary gland organoids induced by inflammatory substances, dexamethasone restored acinar markers such as *AQP5* gene expression levels, while inhibiting pro-inflammatory cytokines *TNF* and *IL6*, as well as chemokines *CCL2*, *CXCL5*, and *CXCL12* induction. Macrophages cultured in inflammatory substance-treated media from salivary gland organoid cultures exhibited pro-inflammatory polarization. However, treatment with dexamethasone shifted them towards an anti-inflammatory phenotype by reducing M1 markers (*Tnf*, *Il6*, *Il1b*, and *Cd86*) and elevating M2 markers (*Ym1*, *Il10*, *Cd163*, and *Klf4*). However, high-dose or prolonged dexamethasone treatment induced acino-ductal metaplasia and had side effects in both *in vivo* and *in vitro* models.

**Conclusions:**

Our findings suggest the effectiveness of corticosteroids in treating obstructive sialadenitis-induced salivary gland dysfunction by regulating pro-inflammatory cytokines.

## Introduction

Salivary gland (SG) obstruction occurs because of various factors, such as infection, allergy, autoimmune disease, iatrogenic trauma, or radiation exposure (1). It is typically associated with SG dysfunction and is characterized by reduced saliva production, swelling, pain, and inflammation (2). Untreated SG obstruction leads to a decrease in saliva production and impairs antimicrobial function, making patients vulnerable to mucositis, caries, candidiasis, dysphagia, and dysarthria. Surgery such as sialendoscopy is often recommended to address underlying pathologies such as stones and stenosis (3). Following the removal of obstructive factors, conservative interventions such as sialogogues, massage, hydration, and anti-inflammatory medications are necessary to restore function and prevent recurrence.

The relationship between SG obstruction, inflammation, and functional consequences is complex. Inflammation in the salivary ducts causes thickening of the ductal epithelium, leading to impaired salivary excretion. The inflammatory response also induces acino-ductal metaplasia, reducing the quantity of saliva and altering its composition (4). Tissue damage triggers chemokine gradients, and adhesion molecules, which attract macrophages to the sites of injury or infection. Activated macrophages can be categorized into two groups: M1 and M2. M1 macrophages produce pro-inflammatory cytokines and initiate an immune response, whereas M2 macrophages are associated with wound healing and tissue repair through their anti-inflammatory functions (5). Obstructive injury causes an increase in the M1 population of macrophages, resulting in inflammation of the bladder (6), ureters (7), and airways (8). Macrophage infiltration increases after SG duct ligation; however, the precise mechanism has not been elucidated (9).

Glucocorticosteroids are commonly used in clinical practice because of their anti-inflammatory and immunosuppressive properties (10). When corticosteroids such as dexamethasone are combined with irrigation, swollen SG can be relieved (11, 12). However, despite the current use of dexamethasone in treating sialadenitis, the precise mechanisms underlying the action of dexamethasone on SGs should be better understood. Dexamethasone reduces myeloperoxidase activity in the ligated gland and increases salivary flow compared to that in the ligated duct; however, this effect was not statistically significant (10). Furthermore, only the acute damage to SGs was examined. The lack of research on the exact mechanism of corticosteroids in SGs is a significant challenge, and relying solely on empirical knowledge of their mechanisms, side effects, and precautions is risky for patients. Therefore, further investigation is also needed to determine the potential side effects of overusing dexamethasone and ensure the optimal treatment (13).

In this study, we investigated the therapeutic effects of corticosteroids in the treatment of obstructive sialadenitis and their accompanying adverse effects. To simulate SG obstruction in mice, a duct ligation model was used. Ligation of the ducts leads to parenchymal atrophy, acinar cell loss, inflammation, and fibrosis in downstream SGs (14). Furthermore, we established an inflammatory SG organoid model using inflammatory substances (lipopolysaccharide; LPS, and tumor necrosis factor; TNF) based on our previous SG organoid culture system (15). This inflamed organoid model enabled us to investigate alterations in SG epithelial cells and immune modulation, including chemotaxis. The overarching goal was to identify the precise mechanism of action of corticosteroids to enable evidence-based treatment decisions. Bridging this research gap is essential, not only for a deeper understanding of commonly used drugs but also to ensure safer and more effective treatment strategies for patients with SG disorders.

## Materials and methods

### Obstructive sialadenitis mouse model

Adult female C57BL/6 mice (8–14 weeks old; Orientbio, Seongnam, Republic of Korea) were maintained at 22 ± 2°C and 50 ± 10% relative humidity on a 12-h light/dark cycle (8 a.m. – 8 p.m.), fed, and provided water ad libitum under specific pathogen-free conditions in a facility accredited by AAALAC International (# 001071). All experiments conducted in this study were approved by the Institutional Animal Care and Use Committee (approval number # 2023–0088) of Yonsei University College of Medicine. Mice were randomly divided into each group as follows: 1) The Sham group was a group with only incision; 2) the DEX group was administered dexamethasone after the deligation; 3) the Ligation group was not deligated after ligation; 4) the Deligation+PBS group was deligated two weeks after ligation and administered with PBS; and 5) the Deligation+DEX group was administered with dexamethasone after the clips that tied the duct for two weeks were removed. To confirm the side effects of dexamethasone, mice were divided into four groups: 1) DEX group administered with dexamethasone only; 2) Deligation+PBS group administered PBS after deligation; 3) Deligation+DEX group administered dexamethasone after deligation; 4) Deligation+DEX^Hi^ group infused with a 10-fold higher concentration of dexamethasone to assess high-dose side effects. To investigate the long-term effect of dexamethasone, the Deligation+DEX^L-T^ group received six administrations of the normal concentration and harvested at 16w. To serve as a long-term control, 16-week normal mice were harvested and analyzed together.

### Duct ligation and dexamethasone administration

For duct ligation, mice were anesthetized via intraperitoneal injection of ketamine (50 mg/kg, Virbac) and rompun (5 mg/kg, Bayer HealthCare, Mississauga, Ontario, Canada). After fixing the mice with medical tape, povidone was spread into the front of the neck. A 1–2 cm incision was made to expose the submandibular glands (SMGs). The left excretory duct of SMG and the belonging nerve and vessels were clipped with a titanium hemostatic clip 9 (3.6 mm, # J9180, VITALITEC, Bargheim, Germany). The incision was closed using Reflex Wound Clips (7 mm, # 203–1000, CellPoint Scientific, Gaithersburg, MD, USA). After 2 weeks, the excretory duct was deligated by removing the clip, except in the Ligation group. Following an *in vivo* methodology (16), either dexamethasone (# D8893–1MG, Sigma-Aldrich, St. Louis, MO, USA) for the Deligation+DEX group or PBS for the Deligation+PBS group was retroductally administered through a cannula implanted in the submandibular duct orifice at 1 μg/20 μL for 10 μL/min. There were three control groups: Sham (incision only), DEX (no ligation and dexamethasone administration), and Ligation (ligation only).

To confirm the side effects of high-dose or long-term dexamethasone use, the mice were further divided into the Deligation+DEX^Hi^ and Deligation+DEX^L-T^ groups. The submandibular duct was ligated and deligated in the same manner. For the DEX^Hi^ group, 10 μg/20 μL dexamethasone was administered. Except for the DEX^L-T^ group, all mice were euthanized 2 weeks after deligation and dexamethasone administration. The DEX^L-T^ group received 1 μg/20 μL of dexamethasone once a week for 6 weeks and were euthanized 1 week after the final administration. As for the control group of the DEX^L-T^ group, normal SGs from 16-week-old mice were harvested at the same time period as the DEX^L-T^ group.

### *In vivo* functional analysis

The mice were euthanized using a CO^2^ chamber, and the left SMG was excised, weighed, and compared between the groups. Prior to euthanasia, mice were intraperitoneally injected with pilocarpine (5 mg/kg, # P6503; Sigma-Aldrich) to induce salivation. The lag time for salivation was measured after pilocarpine administration. 5 min after stimulation, saliva was extracted from the floor of the mouth for 5 min using a micropipette. The collected saliva was placed in 1.7 mL microtubes, and the volume was measured. Lag time was defined as the time of the first salivation following pilocarpine stimulation.

### Histology

The fixed SMG tissues were embedded in paraffin, sectioned, deparaffinized, and hydrated. For histological analysis, the sections were stained with hematoxylin and eosin (H&E) (# ab2455880, Abcam, Cambridge, UK). Periodic acid-Schiff (PAS, # ab150680, Abcam) was utilized to detect mucin secretion, while Masson’s trichrome (MTC, # ab150686, Abcam) was used to stain the collagen fibers. All staining procedures were conducted following the manufacturer’s instructions. A blinded examiner evaluated pathological changes, including inflammation (H&E), mucin production (PAS), and fibrosis (MTC). The damage score of the SMG tissues was recorded as 0–5 based on the following criteria: intact acini in image fields were scored as 0 for 90% or more, 1 for 70%–90%, 2 for 50%–70%, 3 for 30%–50%, 4 for 10%–30%, and 5 for 10% or less. The mucin area was magenta on PAS, and the fibrotic area was blue on MTC. The data were analyzed using ImageJ software (NIH, Bethesda, MD, USA).

### Quantitative real-time PCR

Following the manufacturer’s protocols, total RNA was extracted using TRIZOL (# 15596018, Thermo Fisher) and reverse transcribed into cDNA using the RT Master MIX (# RRO36A, Takara, Kusatsu, Japan). Gene-specific PCR products were quantified using a SYBR reporter and measured with a QuantStudio 5 real-time PCR system (Thermo Fisher). The ΔΔCt method was used to measure gene expression after normalization to housekeeping Gapdh expression. Primer information is presented in **Supplementary Table 1**.

### Immunohistochemical and immunofluorescence staining and image acquisition

For Immunohistochemistry, SMG tissues were cut into 5-μm-thick paraffin blocks and soaked in xylene and a series of alcohols (from 100% to 70%). The sections were subjected to antigen retrieval for 40 min in Tris-EDTA (pH 9.0). Blocking, secondary antibodies, and an avidin/biotin-based peroxidase system were utilized with the ABC-HRP Kit (# PK-6200, VECTASTAIN, Newark, CA, USA). Primary antibodies against TNF (# NBP1–19532, 1:200; NOVUS, Centennial, CO, USA), IL-1β (# NB600–633,1:200; NOVUS), F4/80 (# A18637, 1:100; Abclonal, Woburn, MA, USA), and Ly-6G (# A20861, 1:50; Abclonal) were applied. DAB Quanto (# 12623957, Epredia) was employed to expedite the peroxidase system and its reaction product turned brown.

For immunofluorescence (IF), sections were blocked with 5% normal serum for 1 h at 20–25°C and then incubated with primary antibodies overnight at 4°C. The next day, the sections were incubated with secondary antibodies for 1 h at RT. The slides were counterstained with DAPI and then mounted using a ProLong glass antifade mountant (# P36985, Invitrogen, Carlsbad, CA, USA). Detailed antibody information is presented in **Supplementary Table 2**.

Six to eight images per group were randomly acquired using an Eclipse Ti–U2 inverted microscope (Nikon, Tokyo, Japan) and analyzed using NIS-Elements BR (Nikon). Data were quantified using ImageJ software. Images obtained using the same microscope were equally adjusted for thresholding, and the percentage of the total tissue area positive for a specific marker was calculated and normalized to nuclei area using ImageJ software.

### Human SG organoid culture and inflamed SG organoid models

Human parotid gland biopsies were acquired from individuals with benign SG tumors who provided informed consent and approval from the Institutional Review Board of Yonsei University Severance Hospital (permission number #2017–0226–001). The tissues obtained were used to generate human SG organoids as previously reported (15). Briefly, the tissues were initially sectioned with a blade and then subjected to enzymatic dissociation using collagenase type II (# 17101015, Thermo Fisher Scientific, Waltham, MA, USA) for 1 h, followed by a 10 min incubation with TrypLE Express (# 12604013, Thermo Fisher Scientific). Cells were passed through a 70-μm strainer and embedded in growth factor-reduced Matrigel (# 356231, Corning, Corning, NY, USA). The cells were then grown in GEM media containing Advanced DMEM/F12 (# 12634010, Thermo Fisher) supplemented with 10 mM HEPES (# 15630080, Thermo Fisher), 1 × GlutaMAX (# 35050061, Thermo Fisher), 0.2 μg/mL Primocin (# ant-pm, Invivogen, San Diego, CA, USA), 1× B-27 (# 17504044, Thermo Fisher), 1.25 mM N-acetyl-cysteine (# A9165, Sigma), 1% homemade RSPO1-CM, 100 ng/mL noggin (# 6057-NG, R&D, Minneapolis, MN, USA), 5 ng/mL NRG1 (# 100–03, Peprotech, Cranbury, NJ, USA), 5 ng/mL FGF2 (# 100–18B, Peprotech), 10 ng/mL FGF10 (# 100–26, Peprotech), 5 μM A83–01 (# 2939, Tocris, Abindon, UK), 10 mM niacinamide (# N0636, Sigma), 3 μM prostaglandin E2 (# 2296, Tocris), and 1 μM CHIR99021 (# 2520691, Biogems, Colinas, CA, USA). For the first 2–3 days, 10 μM Y-27632 (# 1254, Tocris) was added to the growth medium.

To investigate the effect of dexamethasone, the organoid groups were divided into four: untreated (None), dexamethasone-only administration (DEX), inflammatory substances (I.S) administration, and I.S. administration after dexamethasone (DEX+I.S). To induce inflammatory responses in the organoids, dexamethasone (1 μM) was added to the media on day 4, followed by agitation to ensure thorough mixing. After 2 h, 10 μg/mL LPS (# L3021, Sigma) and 10 ng/mL TNF (# 300–01A, Peprotech) were added and mixed well, and each group of organoids was harvested 72 h later. To assess the potential side effects of high concentration or prolonged treatment, the high-concentration group (DEX^Hi^+I.S) received 10 μM dexamethasone, representing a concentration 10 times higher than the conventional dexamethasone dosage (1 μM). The long-term treatment group (DEX^L-T^+I.S) was harvested on day 12 and compared to DEX+I.S, which was removed after 3 days, and DEX^L-T^+I.S, which received 1 μM dexamethasone continuously until day 12.

### Mouse bone marrow-derived macrophage culture and organoid cultured medium treatment

Mouse BMDMs were extracted from the tibia and femur of 8-week-old female C57BL/6 mice. Bone marrow cells were collected and cultured on a suspension plate in DMEM (# L0103–500, Biowest, France) supplemented with 10% FBS (# 16000044, Thermo Fisher), 1% P/S (# 15140122, Thermo Fisher), and 20 ng/mL murine M-CSF (# 315–02, Peprotech). The medium was replaced every 2–3 days.

Media from each of the four SG organoid groups (None, DEX, I.S, and DEX+I.S), as well as the inflamed organoids, were harvested and stored at -20°C. We labeled the media in the None, DEX, I.S, and DEX+I.S group media as CM1, CM2, CM3, and CM4, respectively. The group that did not treat CM was labeled Ctrl. Seven days after starting the mouse BMDM culture, media mixed with BMDM culture media and organoid CM at a 1:1 ratio were added. For the polarization to M1 macrophages, LPS and IFNγ (# 575302, Biolegend) were added to the media, while IL4 (# 214–14, Peprotech) and IL13 (# 210–13, Peprotech) were employed to polarize M2 macrophages. The mouse BMDMs were harvested after 24 hours of stimulation for RNA extraction.

### Statistics

All data were analyzed using GraphPad Prism (GraphPad Software ver. 10, San Diego, CA, USA). We used the Shapiro-Wilk test to check the normality of the data. If the data displayed a normal distribution, we employed t-tests for two groups and one-way ANOVA for more than three groups. Tukey’s *post hoc* test was conducted to compare values and determine statistical significance. In some data that did not demonstrate a normal distribution, the Kruskal-Wallis H test, followed by Dunn’s test, was performed for multiple group comparisons. We considered * p < 0.05, ** p < 0.01, and *** p < 0.001 as the significance levels. Further details are provided in the figure legends.

## Results

### Morphological and functional recovery following dexamethasone treatment after deligation

**Figure 1:**
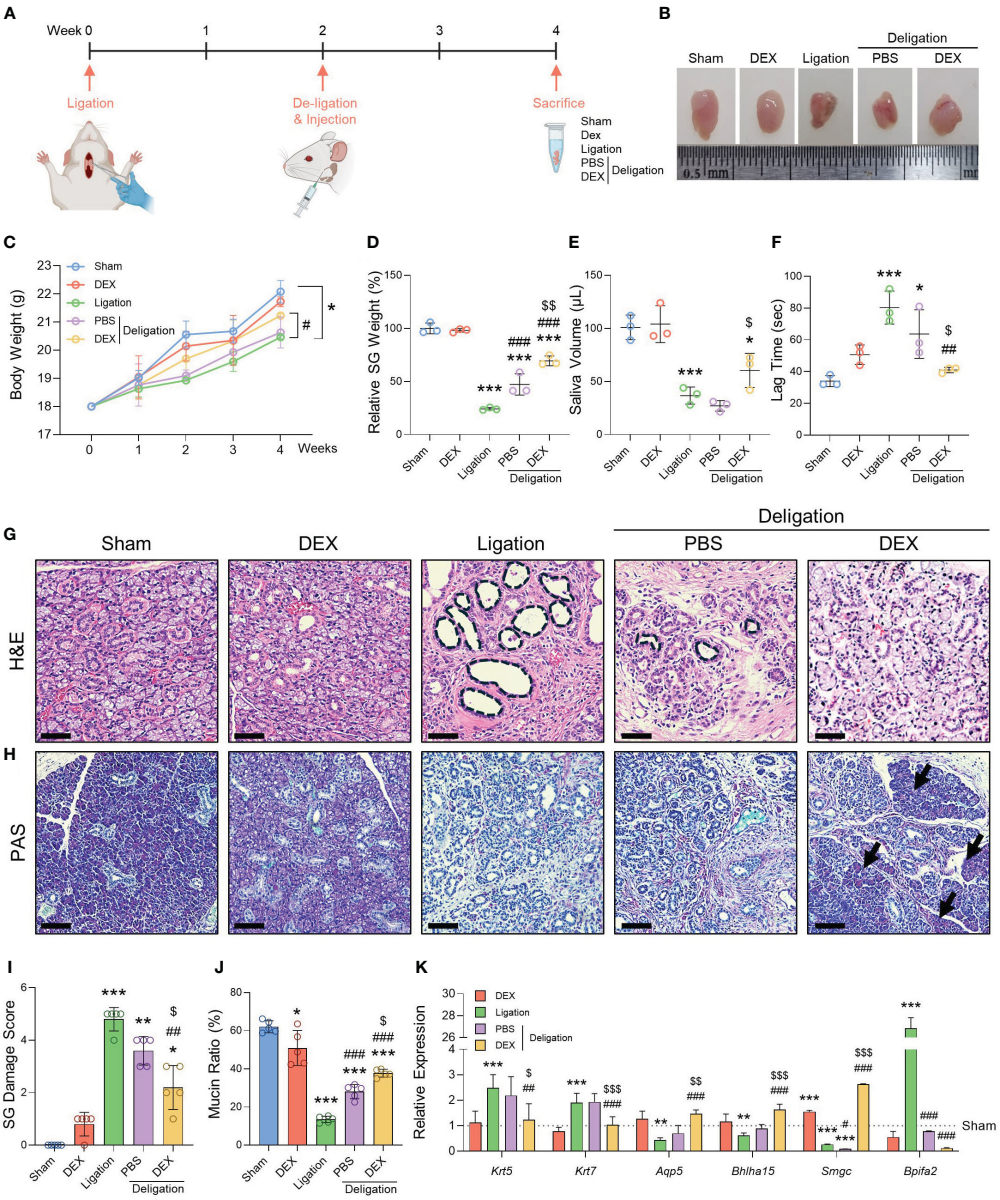
Histological and functional changes after ligation and dexamethasone treatment. **(A)** Graphics were created with BioRender.com. **(B)** A representative macroscopic image of SMGs at week 4. **(C)** Body weight was measured once a week for 4 weeks. **(D)** Relative SG weight was assessed. **(E)** Saliva volume post-pilocarpine stimulation, and **(F)** lag time to salivation were obtained at week 4. Histological images stained with **(G)** H&E and **(H)** PAS. Notable features such as dilated ducts (black asterisks) and recovered acinus (black arrows) are indicated. Scale bar = 50 μm. **(I)** The SG Damage Score was determined by assessing the morphological breakdown of acinar and ductal structures as observed following H&E staining. The Kruskal-Wallis H test was used to analyze the SG Damage Score data, followed by Dunn’s test for multiple group comparisons. **(J)** The mucin ratio was measured by the magenta areas after PAS staining. N = 3~5 mice per group were used. The densitometric analysis involved 6~8 random field selections, with computations performed using Image J software. The data are presented as the mean ± SD. **(K)** qRT-PCR analysis of the gene markers *Krt5, Krt7, Aqp5, Bhlha15*, *Smgc*, and *Bpifa2*. Gene expression was normalized relative to the expression of Gapdh. Three independent experiments for qRT-PCR were conducted. Data are presented as mean ± SD. A one-way ANOVA with Tukey’s *post hoc* test was used to compare groups on data excluding the SG Damage Score data; * compared with Sham; # compared with Ligation; $ compared with Deligation+PBS. * p < 0.05, ** p < 0.01, *** p < 0.001, ## p < 0.01, ### p < 0.001, $ p < 0.05, $$ p < 0.01, and $$$ p < 0.001.

First, we investigated the therapeutic effects of dexamethasone on obstructive sialadenitis using *in vivo* duct ligation and deligation models. To induce severe obstruction, we ligated the nerves and vessels along the submandibular duct based on our previous protocol (15). Duct ligation was performed at week 0, deligation was conducted 2 weeks after ligation, and SMGs were extracted 2 weeks after retroductal administration of either dexamethasone or PBS following deligation (**Figure 1A**). Ligation damage induced SG atrophy in the Ligation group; however, the SGs in the Deligation+DEX group returned to a size similar to that in the Sham group (**Figure 1B**). The body weight slowly increased in the duct-ligated groups (Ligation, Deligation+PBS, and +DEX) and tended to recover after deligation. Only the Deligation+DEX group showed a more significant recovery in body weight than the Ligation group (**Figure 1C**).

To determine whether the administration of dexamethasone facilitated the recovery of function after deligation, we measured SG weight, saliva volume, and lag time to salivation. The Ligation group had a significantly lower SG weight than the Sham group (23%). However, the Deligation+PBS group showed a recovery in gland weight of up to 47%, and the Deligation+DEX group showed a recovery of up to 70% compared with the Sham group (**Figure 1D**). Saliva volume was measured for 5 min, and only the Deligation+DEX group showed an increase in saliva volume compared to the Ligation group. No significant changes were observed in the Deligation+PBS group (**Figure 1E**). There was considerable lag time in the Ligation group, indicating a lack of salivation, which significantly recovered after deligation and dexamethasone treatment (**Figure 1F**).

To examine the structure of SG, H&E and PAS staining was performed. The duct-ligated groups showed dilated ducts, whereas deligation and dexamethasone administration reduced the diameter of the dilated ducts (**Figure 1G**, black circles). PAS staining revealed a decrease in the Ligation group and an increase in mucin production in the Deligation+DEX groups (**Figure 1H**, arrows). Moreover, the Deligation+DEX group also exhibited the most substantial recovery from SG damage, as evidenced by the morphological improvement in the acinar and ductal structures (**Figures 1G, I**). The magenta area, as assessed by PAS staining, demonstrated an increased mucin production ratio in the Deligation+PBS group. However, dexamethasone treatment resulted in a more significant restoration of mucin production compared to the Deligation+PBS group (**Figures 1H, J**). Gene expression analysis revealed that *Krt5* (basal cell marker) and *Krt7* (luminal cell marker) were upregulated by ligation and downregulation by dexamethasone treatment. Conversely, *Aqp5* (pro-acinar marker) and *Bhlha15* (mature acinar marker) were downregulated following injury but restored by dexamethasone treatment. Similarly, *Smgc* (adult murine submandibular specific mucin marker) exhibited a pattern consistent with the acinar markers, while *Bpifa2* (pro-acinar cell marker) followed a trend similar to the ductal markers (**Figure 1K**). These results suggest that administering dexamethasone retroductally following the relief of SG obstruction promotes both microscopic and functional recovery from SG damage.

### SG cellular changes after ligation and deligation with dexamethasone treatment

**Figure 2:**
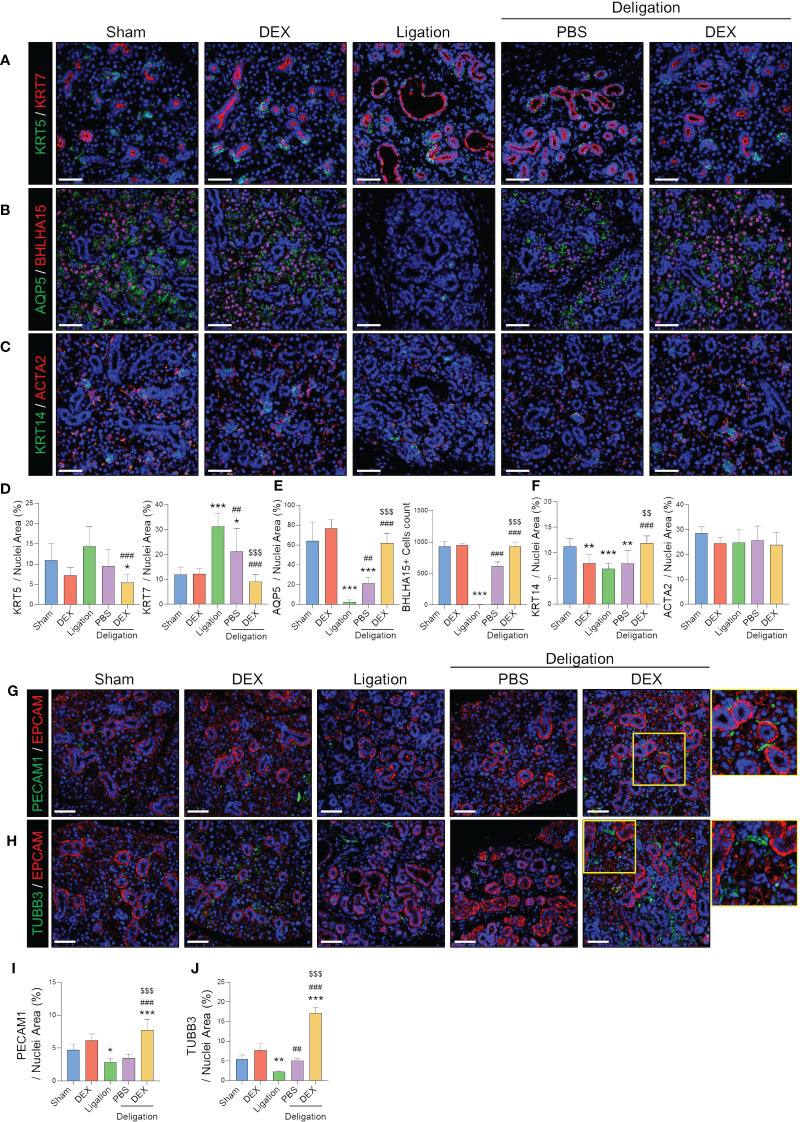
Endothelial and neuronal cells around ductal cells that were completely recovered by dexamethasone treatment. **(A)** Representative IF images of basal (KRT5; green) and luminal (KRT7; red) cells, and **(D)** quantified positive area. **(B)** Representative IF images of pro-acinar (AQP5; green) and mature acinar (BHLHA15; red) cells, and **(E)** quantified the positive area of AQP5 and counts of BHLHA15^+^ cells. **(C)** Representative IF images of basal progenitor (KRT14; green) and myoepithelial (ACAT2; red) cells and **(F)** quantified positive area. **(G)** IF staining was performed using endothelial (PECAM1; green), epithelial (EPCAM; red), and **(H)** neural (TUBB3; green) cells. **(I, J)** A quantified positive area of PECAM1 and TUBB3 was obtained. The nuclei were counterstained with DAPI. Scale bar = 50 μm. N = 3~5 mice per group were used. The densitometric analysis involved 6~8 random field selections, with computations performed using Image J software. The IF quantification was expressed as a percentage by normalizing the positive area by the nuclei area. Data are presented as mean ± SD. A one-way ANOVA with Tukey’s *post hoc* test was used to compare groups; * compared with Sham; # compared with Ligation; $ compared with Deligation+PBS. * *p* < 0.05, ** *p* < 0.01, *** *p* < 0.001, ## *p* < 0.01, ### *p* < 0.001, $$ *p* < 0.01, and $$$ *p* < 0.001.

Following ligation and deligation, we investigated alterations in SG epithelial and stromal cell markers, focusing on the SGs with dexamethasone treatment. We focused on the changes in salivary acinar, ductal, and myoepithelial cells. The count of KRT5^+^ basal cells and KRT7^+^ luminal cells elevated post-injury (**Figures 2A, D**). Dexamethasone administration significantly reduced the KRT7-positive areas, suggesting an improvement in luminal duct dilation. The number of AQP5^+^ pro-acinar cells and BHLHA15^+^ mature acinar cells was dramatically reduced by ligation injury, with barely any visible BHLHA15^+^ cells (**Figure 2B**). However, partial recovery occurred with PBS administration post-deligation, whereas dexamethasone treatment led to almost complete regeneration of AQP5-and BHLHA15^+^ cells (**Figures 2B, E**). KRT14^+^ basal progenitor cells decreased following injury but exhibited a greater increase with dexamethasone than in the Sham condition. However, ACTA2+ myoepithelial cells did not show any difference between groups (**Figures 2C, F**). Our findings suggest that duct ligation mainly affects mucin-secreting acinar and ductal cells rather than myoepithelial cells. Moreover, dexamethasone appears to facilitate the regeneration of salivary epithelial cells following duct ligation.

Furthermore, we observed that PECAM1^+^ endothelial cells were present around the duct and acinar in the Sham group. However, their expression was reduced following ligation-induced damage. Dexamethasone treatment resulted in an increased number of PECAM1^+^ cells along the duct (**Figures 2G, I**). In addition, dexamethasone administration significantly increased the number of TUBB3^+^ neurons (**Figures 2H, J**). Among the control groups, there was a slight increase in PECAM1^+^ and TUBB3^+^ areas in the DEX group, although the difference was not significant (**Figures 2I, J**). These results suggest that dexamethasone may stimulate SG stromal cells during the repair of damaged SG epithelial cells.

### Cytokine changes after dexamethasone treatment regulated the infiltration of macrophages in SGs

**Figure 3:**
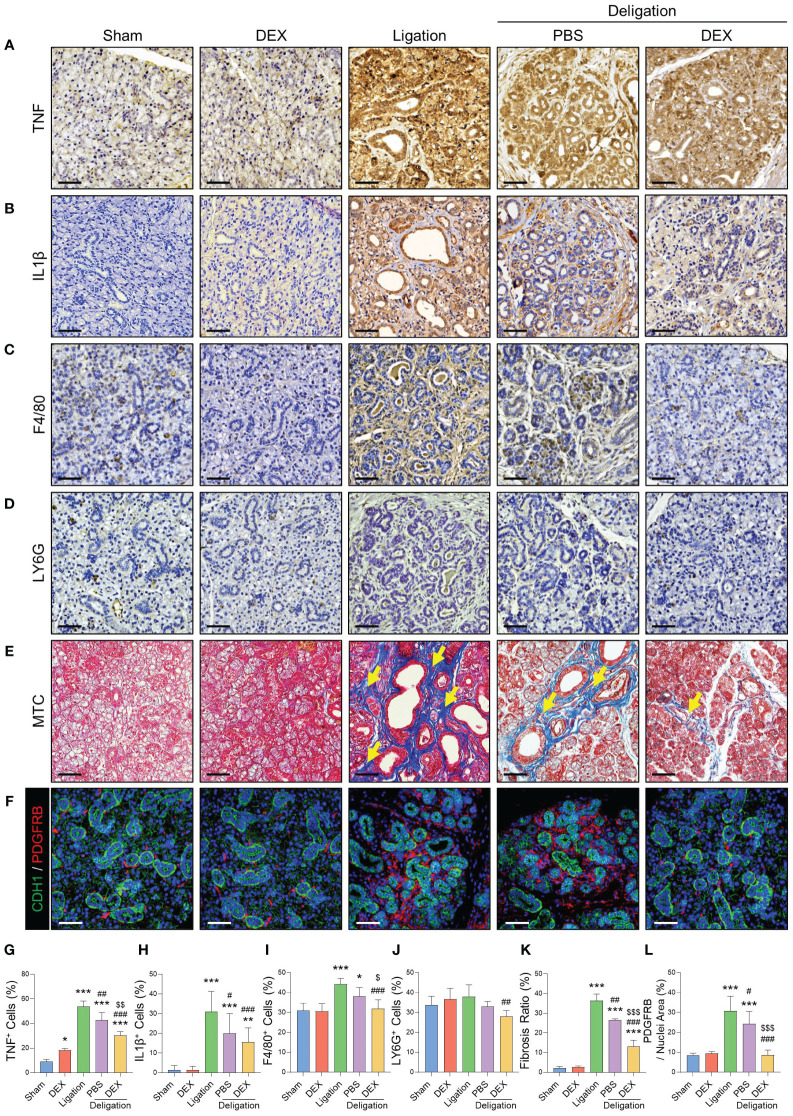
Macrophages recruited by inflammatory cytokines exacerbated the fibrosis, which dexamethasone reduced. Immunohistochemistry staining was conducted to detect TNF **(A)**, IL-1β **(C)**, F4/80 **(E)**, and LY6G **(G)**. Images were quantified based on the staining intensity of positive areas. Quantifications of TNF **(B)**, IL-1β **(D)**, F4/80 **(F)**, and LY6G **(H)**. **(I)** MTC staining images were obtained using an Eclipse Ti–U2 inverted microscope, and the fibrotic area (yellow arrows) is indicated. **(J)** Fibrosis ratio was assessed using blue fibrotic areas after MTC staining. **(K)** IF staining was performed for epithelial (CDH1; green) and stromal (PDGFRB; red) cells, and **(L)** quantified positive areas for PDGFRB. Scale bar = 50 μm. N = 3~5 mice per group were used. The densitometric analysis involved 6~8 random field selections, with computations performed using Image J software. The IF quantification was expressed as a percentage by normalizing the positive area by the nuclei area. Data are presented as the mean ± SD. A one-way ANOVA with Tukey’s *post hoc* test was used to compare groups; * compared with Sham; # compared with Ligation; $ compared with Deligation+PBS. * *p* < 0.05, ** *p* < 0.01, *** *p* < 0.001, # *p* < 0.05, ## *p* < 0.01, ### *p* < 0.001, $ *p* < 0.05, $$ *p* < 0.01, and $$$ *p* < 0.001.

To analyze how dexamethasone ameliorates salivary inflammation and promotes recovery, we measured the expression of TNF and IL-1β using Immunohistochemistry. These pro-inflammatory cytokines increase under inflammatory conditions (17). The expression levels of TNF and IL-1β were significantly increased by salivary duct ligation (**Figures 3A, B, G, H**). We examined whether the recruitment of macrophages resulted from cytokine chemotaxis. F4/80^+^ macrophages exhibited a 13% increase in the Ligation group compared to the Sham group, while the Deligation+DEX group exhibited a significant reduction to 32% of the macrophage percentage, approaching normal levels (**Figures 3C, I**). However, no significant changes were observed in the LY6G^+^ neutrophil population. (**Figures 3D, J**).

We next explored the changes in macrophages that showed significant change (**Figures 3C, I**). Myofibroblasts, major contributors to fibrosis, become activated through autocrine and paracrine signaling from macrophages (18). Macrophages can trigger fibroblast activation and pro-inflammatory activity during tissue repair (19). MTC staining indicated a statistically significant reduction in the percentage of fibrotic cells with dexamethasone treatment (**Figures 3E**, **K**). As the increase in ACTA2 expression was not significant (**Figures 2C, F**), we conducted IF staining for PDGFRB, a marker of salivary stromal fibroblasts, to explore the involvement of other stromal cell subtypes in ligation-induced fibrosis. The results revealed a significant increase in the number of PDGFRB^+^ cells post-ligation, followed by a significant decline during recovery, consistent with the MTC findings (**Figures 3F, L**). This finding suggests that macrophages, recruited via chemoattraction during ductal ligation, and fibrosis, induced by ligation were relieved by dexamethasone.

### The inflammatory substances-induced SG inflammation organoid model was established to investigate the mode of action of dexamethasone against SG inflammation

**Figure 4:**
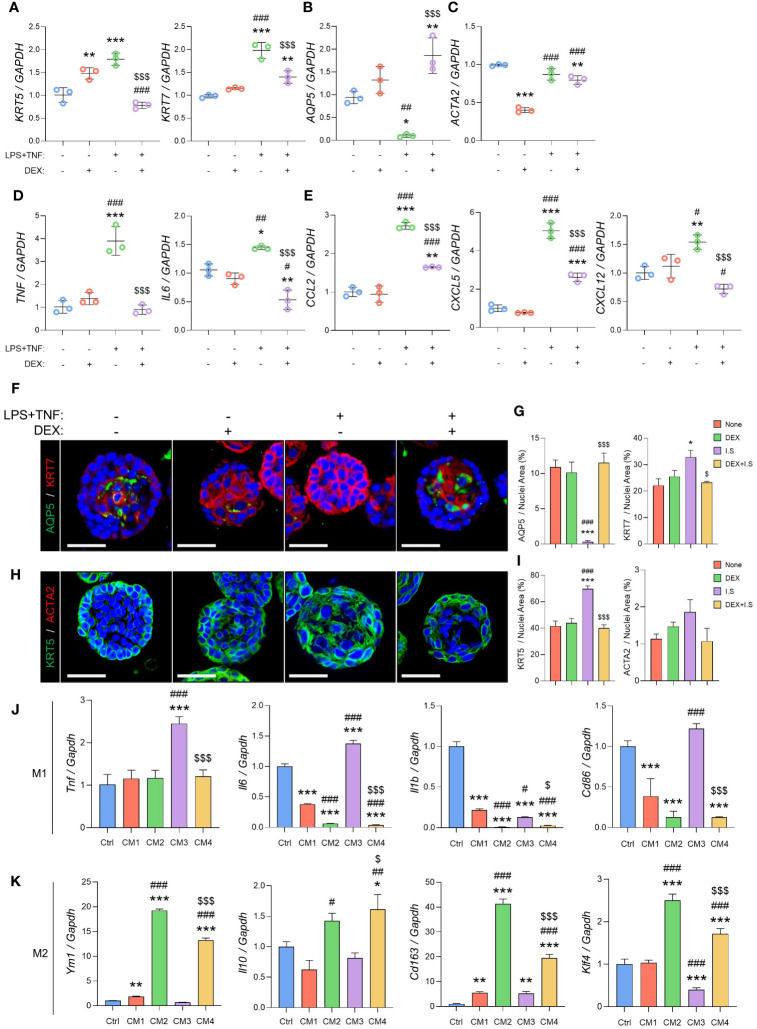
Inflammation causes acino-ductal metaplasia in SG epithelial cells, resulting in a loss of functionality. The inflamed organoids were treated with dexamethasone. After harvesting, the organoids were subjected to qRT-PCR to evaluate gene expression. **(A)** Expression of ductal cell markers *KRT5* and *KRT7*. **(B)** Expression of the acinar cell marker *AQP5*. **(C)** Expression of the myoepithelial cell marker *ACTA2*. **(D)** Expression of the pro-inflammatory cytokine markers *TNF* and *IL6*. **(E)** Expression of chemokine markers *CCL2*, *CXCL5*, and *CXCL12*. Gene expression was normalized to that of *GAPDH*. A one-way ANOVA with Tukey’s *post hoc* test was used to compare groups; * compared with None; # compared with DEX; $ compared with DEX+I.S. N = 3 per each group. Data are presented as mean ± SD. A one-way ANOVA with Tukey’s *post hoc* test was used to compare groups; * *p* < 0.05, ** *p* < 0.01, *** *p* < 0.001, # *p* < 0.05, ## *p* < 0.01, ### *p* < 0.001, and $$$ *p* < 0.001. **(F)** Representative IF images of pro-acinar (AQP5; green) and luminal (KRT7; red) cells, and **(G)** quantified positive areas. **(H)** Representative IF images of basal (KRT5; green) and myoepithelial (ACTA2; red) cells, and **(I)** quantified positive areas. Scale bar = 50 μm. N = 3~5 mice per group were used. The densitometric analysis involved 6~8 random field selections, with computations performed using Image J software. The IF quantification was expressed as a percentage by normalizing the positive area by the nuclei area. Data are presented as the mean ± SD. A one-way ANOVA with Tukey’s *post hoc* test was used to compare groups; * compared with None; # compared with DEX; $ compared with I.S. * *p* < 0.05, *** *p* < 0.001, ### *p* < 0.001, $ *p* < 0.05, and $$$ *p* < 0.001. **(J)** Expression of M1 macrophage markers including *Tnf, Il6, Il1b*, and *Cd86*, and **(K)** M2 macrophage markers such as *Ym1*, *Il10*, *Cd163*, and *Klf4*. Gene expression was normalized to that of *Gapdh*. Three independent experiments for qRT-PCR were conducted. Data are presented as mean ± SD. A one-way ANOVA with Tukey’s *post hoc* test was used to compare groups; * compared with Ctrl; # compared with CM1; $ compared with CM3. * *p* < 0.05, ** *p* < 0.01, *** *p* < 0.001, # *p* < 0.05, ## *p* < 0.01, ### *p* < 0.001, $ *p* < 0.05, and $$$ *p* < 0.001.

Our *in vivo* experimental design aimed to assess the impact of dexamethasone over 2 weeks to ensure significant damage and subsequent recovery. Because of the short half-life of dexamethasone (36–54 h), we developed a 3-dimensional organoid culture system to efficiently investigate the underlying mechanism (20). After 3 days of growth, the organoids were treated with LPS (10 µg/mL) and TNF (10 ng/mL) to induce inflammation, while dexamethasone (1 µM) was administered to assess its anti-inflammatory effects. Treatment with inflammatory substances resulted in increased *KRT5* and *KRT7* expression and decreased expression of the acinar markers *AQP5* (**Figures 4A, B**). However, *ACTA2* did not exhibit inflammation-dependent changes (**Figure 4C**). In the inflamed organoid model, the mRNA levels of ductal, acinar, and myoepithelial cell markers in the DEX+I.S group fully recovered to the expression levels of the None group, closely matching the results of the *in vivo* experiments (**Figures 4A–C**). Additionally, we analyzed the cytokine genes to study the immunoregulatory mechanisms of SG epithelial cells. For cytokines, we used the inflammatory cytokines *TNF* and *IL6* as markers. For chemokines, we used *CCL2*, which attracts monocytes/macrophages in inflammatory situations; *CXCL5*, which recruits leukocytes, especially neutrophils; and *CXCL12*, a chemokine secreted in the presence of inflammatory stimuli such as LPS, TNF, or IL1. The results demonstrated a significant increase in immune-related gene expression in epithelial cells due to inflammation, including elevated levels of *TNF* and *IL6* (**Figure 4D**) and *CCL2*, *CXCL5*, and *CXCL12* (**Figure 4E**), which were subsequently restored to normal levels with dexamethasone treatment. Furthermore, IF staining demonstrated that the inflammatory substances reduced the expression of AQP5 and increased KRT7 levels, while treatment with dexamethasone reversed these effects, similar to the None group (**Figures 4F, G**). Thus, KRT5 levels in IF were increased in response to inflammation and decreased by treating with dexamethasone, whereas ACTA2 showed no significant difference (**Figures 4H, I**). These results are in agreement with the *in vivo* IF results in **Figures 2A–F**, demonstrating that the investigations of dexamethasone through organoid inflammation modeling can be representative of *in vivo* biological changes.

To assess the impact of cytokines and chemokines secreted by epithelial cells on macrophages, we exposed macrophages to an organoid CM to evaluate macrophage polarization. M1 macrophage markers with pro-inflammatory roles include the cytokines *Tnf*, *Il6*, *Il1b*, and *Cd86*, which are involved in antigen presentation (21). Upon induction with M1 macrophages, there was a significant increase in M1 markers in the M1+CM3 group, which consisted of an organoid CM treated with inflammatory substances. After the induction of inflammation, the M1+CM4 group treated with dexamethasone showed a significant decrease in both markers (**Figure 4J**). For M2 macrophage markers, we used *Ym1*, which plays a role in macrophage activation; *Il10*, an immunosuppressive cytokine; *Cd163*, a scavenger receptor for monocytes/macrophages; and *Klf4*, which regulates M2 polarization (21–24). In CM2, the medium of organoids treated with dexamethasone, all M2 markers were upregulated. In the M2+CM3 group, most showed significantly lower expression compared to the M2+CM1 group. The M2+CM4 group also showed an increase in M2 marker levels, similar to those in the M2+CM2 group (**Figure 4K**). Inflammatory organoids accurately replicated epithelial conditions *in vivo*, thereby facilitating mechanistic studies. Furthermore, we demonstrated that cytokines and chemokines released by epithelial cells strongly regulated macrophage polarization, and that epithelial cells treated with dexamethasone downregulated M1 macrophages and upregulated M2 macrophages.

### Potential side effects of high-dose or long-term dexamethasone treatment in SGs

**Figure 5:**
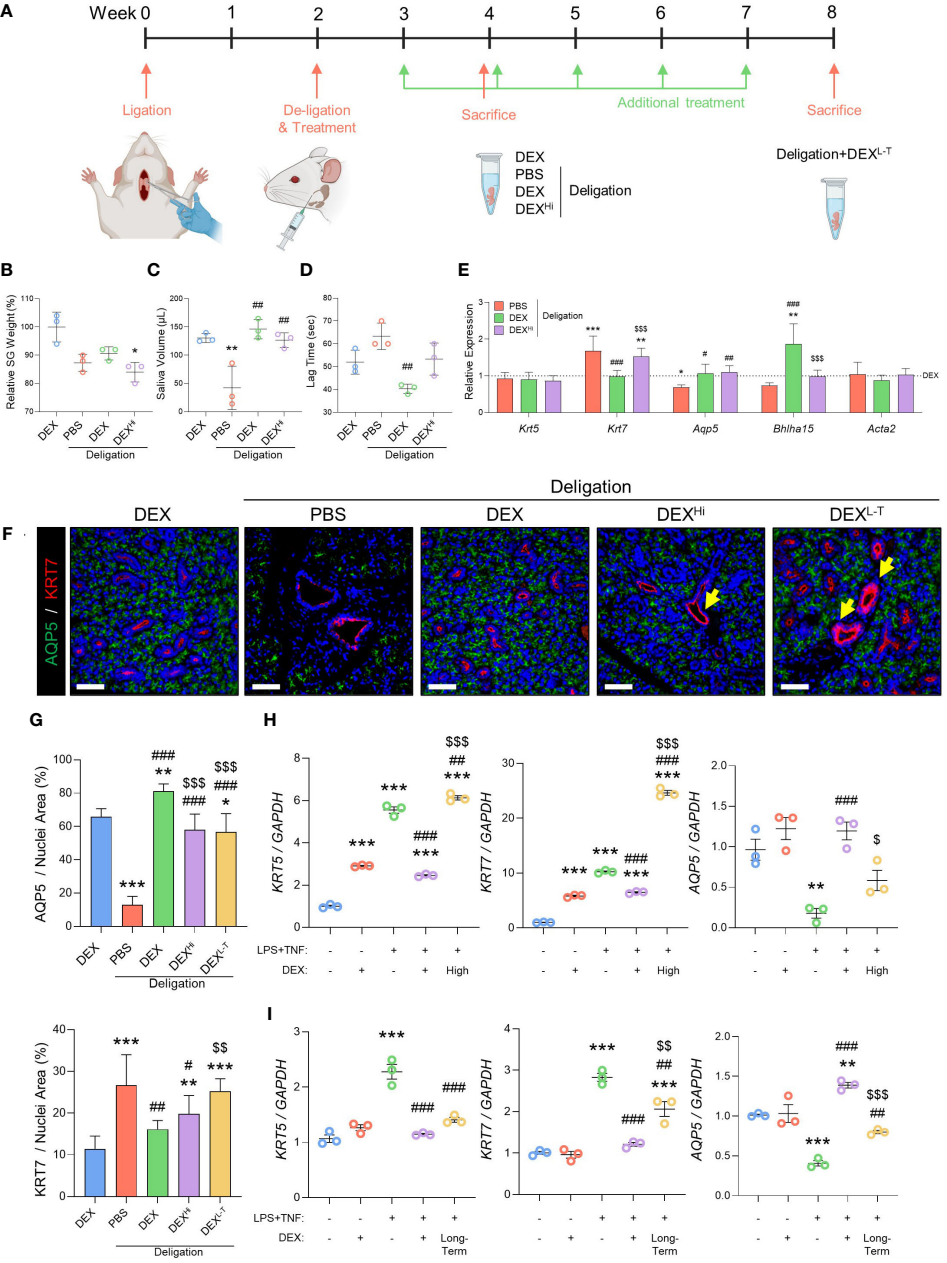
High-dose or long-term administration of dexamethasone prevented the regeneration effect of dexamethasone. **(A)** Graphics were created with BioRender.com. Relative SG weight **(B)**, saliva volume after pilocarpine stimulation **(C)**, and lag time to salivation **(D)** were measured at week 4, but the DEX^L-T^ group was checked at week 8. The gene expression level of *Krt5, Krt7, Aqp5, Bhlha15*, and *Acta2* was measured using qRT-PCR **(E)**. Gene expression was normalized to that of *Gapdh*. N = 3~5 mice per group were used. The densitometric analysis involved 6~8 random field selections, with computations performed using Image J software. Data are presented as mean ± SD. A one-way ANOVA with Tukey’s *post hoc* test was used to compare groups; * compared with DEX; # compared with Deligation+PBS; $ compared with Deligation+DEX. * *p* < 0.05, ** *p* < 0.01, *** *p* < 0.001, # *p* < 0.05, ## *p* < 0.01, ### *p* < 0.001, and $$$ *p* < 0.001. **(F)** Representative IF images of pro-acinar (AQP5; green) and luminal (KRT7; red) cells. Thicken ducts (yellow arrows) are indicated. **(G)** quantified positive areas for AQP5 and KRT7. The densitometric analysis involved 6~8 random field selections, with computations performed using Image J software. The IF quantification was expressed as a percentage by normalizing the positive area by the nuclei area. Data are presented as the mean ± SD. A one-way ANOVA with Tukey’s *post hoc* test was used to compare groups; * compared with DEX; # compared with PBS; $ compared with Deligation+DEX. * *p* < 0.05, ** *p* < 0.01, *** *p* < 0.001, # *p* < 0.05, ## *p* < 0.01, ### *p* < 0.001, $$ *p* < 0.01, and $$$ *p* < 0.001. For the high-dose treatment **(H)** and long-term treatment **(I)**, in organoid model, qRT-PCR analysis of ductal and acinar markers, *KRT5, KRT7*, and *AQP5* Gene expression was normalized relative to the expression of *GAPDH*. Three independent experiments for qRT-PCR were conducted. Data are presented as mean ± SD. A one-way ANOVA with Tukey’s *post hoc* test was used to compare groups; * compared with None; # compared with I.S; $ compared with DEX+I.S. * *p* < 0.05, ** *p* < 0.01, *** *p* < 0.001, # *p* < 0.05, ## *p* < 0.01, ### *p* < 0.001, $ *p* < 0.05, $$ *p* < 0.01 and $$$ *p* < 0.001.

Next, we assessed the side effects of dexamethasone when used at high doses or in the long term. These experiments involved the addition of two new groups: DEX^hi^ and DEX^L-T^. The high-dose group (DEX^Hi^) received a 10-fold higher concentration (10 μg/20 μL) than the DEX group, and the long-term group (DEX^L-T^) received dexamethasone once a week for a total of six doses, with each dose consisting of 1 μg/20 μL. **Figure 5A** shows the experimental design used to test the side effects of dexamethasone *in vivo*. High-dose administration of dexamethasone resulted in significantly lower gland weights (**Figure 5B**). Saliva volume was assessed to verify the effect of dexamethasone, and no significant differences were found in the Deligation+DEX^Hi^ (**Figure 5C**). The lag time tended to increase again in the Deligation+DEX^Hi^ group compared to the Deligation+DEX group, but statistical significance is not observed due to the variation in values (**Figure 5D**). Gene expression analysis revealed that the genes restored to the expression pattern of the DEX group by dexamethasone treatment deteriorated in the Deligation+DEX^Hi^ group. Specifically, *Krt7* increased, and *Aqp5* and *Bhlha15* decreased. *Krt5* and *Acta2* showed no change (**Figure 5E**). In the Deligation+DEX^L-T^ group, there was no difference in SG weight compared to the 16w Normal group, but there was a significant reduction in saliva volume and a significant increment in lag time (**Supplementary Figures S1A–C**). Gene expression analysis of the Deligation+DEX^L-T^ group showed a significant increase in *Krt7* and *Acta2* and a statistically significant decrease in *Aqp5* and *Bhlha15* but no change in *Krt5* (**Supplementary Figure S1D**). In the Deligation+DEX group, both KRT7 and AQP5 exhibited recovery patterns similar to those observed in the DEX group. However, in the side effects group, elevated KRT7 levels were observed owing to duct dilation. Notably, the KRT7 layer showed thickening, and the presence of acinar loss around the ducts, as indicated by yellow arrows (**Figure 5F**). As indicated in the quantification graph, a significant decrease in AQP5 was observed in the DEX^Hi^ and the DEX^L-T^ group compared to the Deligation+DEX group, while KRT7, which was decreased in the Deligation+DEX group, was rebounded by long-term usage of dexamethasone (**Figure 5G**). This suggests the occurrence of acino-ductal metaplasia in the high-dose and long-term groups.

We performed the SG organoid experiments to confirm the adverse effects of dexamethasone. The high-dose group was treated with dexamethasone (10 µM), and the long-term group, which was treated with dexamethasone for 9 days, showed markedly increased expression of *KRT5* and *KRT7* and significantly reduced expression of *AQP5* (**Figures 5H, I**). In conclusion, high-dose, long-term administration of dexamethasone caused acino-ductal transitions. Therefore, prescribing dexamethasone for an extended period may cause adverse effects during recovery from SG dysfunction.

## Discussion

In this study, we aimed to investigate the anti-inflammatory properties of dexamethasone on obstruction-induced sialadenitis and SG fibrosis, as well as the potential side effects associated with dexamethasone overuse. Dexamethasone demonstrated strong anti-inflammatory effects in murine models of ductal obstruction and inflamed SG organoids by altering the environment of epithelial cells. Dexamethasone not only showed significant functional recovery but also exhibited effectiveness in both epithelial and non-epithelial cell populations. In our SG organoid model, dexamethasone can effectively regulate the secretion of cytokines and chemokines from epithelial cells to control the differentiation of macrophages. However, the prolonged or high-dose treatment showed potential adverse effects, leading to acino-ductal metaplasia in both *in vivo* and *in vitro* models (**Figures 5E–I**).

PDGFRB is involved in vascular development under homeostatic conditions, whereas in the context of injury, it plays a role in fibrosis, particularly in renal (25), kidneys (26), and SGs (27). Our study revealed that fibrosis from SG duct ligation was primarily due to PDGFRB^+^ stromal fibroblasts rather than ACTA2^+^ myofibroblasts. Interestingly, these results align with a study on SG obstruction models, which did not show a significant change in ACTA2 expression, a classic myofibroblast marker, during fibrosis while there was an increase in PDGFR-alpha and -beta (27).

Recent studies have shown that specific subsets of macrophages in SGs are regulated by Hedgehog signaling and play a role in regulating epithelial progenitor cells (28, 29). We speculate that certain macrophage subsets may also be involved in repairing epithelial cells in duct obstruction injuries. In our previous study (4), we discovered that human SG organoids release chemokines that attract immune cells. Based on these findings, we investigated how inflammatory conditions impact macrophage activity using the SG inflammatory organoid model. When SG organoid-CM was exposed to mouse bone marrow-derived macrophages, we observed an increase in M1 markers when exposed to inflammatory substances (LPS+TNF; I.S)-treated CM but a decrease in M1 markers with dexamethasone-treated CM. In the inflammatory condition with I.S., most M2 markers decreased, whereas they increased when exposed to dexamethasone-treated CM. Generally, M2 macrophages are recognized for their anti-inflammatory functions (30, 31). M2 macrophages include M2a, M2b, M2c, and M2d subtypes, each characterized by different markers and secreted cytokines. Recent studies have shown that the balance between M2a and M2c subtypes influences tissue regeneration or fibrosis (32). M2c macrophages inhibit the transformation of fibroblasts into myofibroblasts by releasing IL10, while M2a macrophages promote fibrosis through TGFβ secretion. Our data indicated increased *IL10* in CM2 and CM4, accompanied by elevated expression of *CD163*, a marker for M2c macrophages. This suggests that dexamethasone treatment boosted IL10 secretion, leading to the differentiation of macrophages into M2c subtypes thereby reducing fibrosis.

In our *in vivo* study, we observed a significant increase in F4/80^+^ macrophages after ligation, accompanied by a rise in fibrosis. This suggests a link between macrophages and fibrosis (33). Additionally, we observed coordinated patterns of TGFβ and macrophage activity, suggesting that the M2a subtype may contribute to fibrosis by releasing TGFβ and promoting fibroblast differentiation in response to ligation injury (data not shown). After treatment with dexamethasone, fibrosis decreased, suggesting that dexamethasone may help reduce fibrosis by regulating macrophage differentiation through interactions with SG epithelial cells and macrophages. Among these interactions, M2c macrophages may inhibit the transformation of fibroblasts into myofibroblasts following dexamethasone treatment, as suggested in our organoid experiment. However, it’s important to note that SG organoids do not represent all aspects of injury, fibrosis, and regeneration in living organisms because fibrosis in living organisms is highly complex and is influenced not only by macrophages, but also by various other cells, primarily myofibroblasts, epithelial cells, and other immune cells (34). Future studies should focus on specific subsets of macrophages affected by dexamethasone and clarify the regulatory mechanisms of fibrosis. Alternative methods for inducing inflammation in organoids, such as micro-laser dissection instead of chemical induction, can be considered to more accurately mimic *in vivo* conditions.

The previous SG duct ligation model only involved ligating the duct and then harvesting the SGs within 1-week post-ligation (35, 36). This made it difficult to detect severe damage. In contrast, our mouse model showed persistent duct dilation, acinar collapse, fibrosis, and macrophage infiltration even after 4 weeks. This suggests that our duct obstruction model more accurately reflects chronic inflammation rather than acute inflammation. It provides a valuable framework for studying prolonged pathological processes in SGs.

In a previous study simulating SG inflammation, organoids treated with TNF exhibited reduced swelling capacity and diminished AQP5 levels (37). To increase the clinical relevance of the model, we co-administered LPS and TNF to activate the pathogen antigen toll-like receptor 4 in organoids. This intervention elicited noteworthy changes in ductal markers and modifications in epithelial cells, thereby facilitating interactions with the immune system. The organoid model is predominantly composed of epithelial cells, allowing researchers to closely examine the cellular and molecular mechanisms affecting SG cells. Additionally, since other cell types, such as stromal cells, are present in very low numbers, we can focus on the critical epithelial components to understand the various states and responses within SG cells. Our observations, including the increased production of cytokines and chemokines by epithelial cells, provide valuable insights into the dynamics of SG inflammation. Furthermore, we found that our organoid model is a good model to look at interactions between macrophages and other immune cells in inflammatory situations.

The anti-inflammatory effects of dexamethasone are well-known in various organs, but there have been limited studies on its effects on salivary glands (38, 39). Our study demonstrated that dexamethasone reduced pro-inflammatory cytokines such as TNF and IL-1β, and helped restore salivary gland function in inflammatory conditions caused by obstruction by modulating macrophages and myofibroblasts. However, our *in vivo* and organoid studies revealed that both overuse and long-term use of dexamethasone led to an increase in ductal markers and a decrease in acinar markers. These results suggest that high-dose and long-term use of dexamethasone may cause acino-ductal metaplasia and reverse salivary function. Further research is needed to understand the molecular mechanisms behind dexamethasone’s effects on acino-ductal metaplasia.

In summary, our proposition suggests that obstructive SG damage is aggravated by PDGFRB-related fibrosis and macrophage activation. Dexamethasone has the potential to restore SG function in inflammatory conditions following obstruction by modulating epithelial cells and surrounding cellular components. However, it is noteworthy that excessive concentrations or prolonged use of dexamethasone may detrimentally affect SG function. Therefore, cautious prescribing practices are essential to achieve optimal therapeutic outcomes.

## Data availability statement

The original contributions presented in the study are included in the article/**Supplementary Material**. Further inquiries can be directed to the corresponding author.

## Ethics statement

The studies involving humans were approved by Institutional Review Board of the Yonsei University Severance Hospital. The studies were conducted in accordance with the local legislation and institutional requirements. The participants provided their written informed consent to participate in this study. The animal study was approved by Institutional Animal Care and Use Committee at Yonsei University College of Medicine. The study was conducted in accordance with the local legislation and institutional requirements.

## Author contributions

SH: Conceptualization, Data curation, Formal analysis, Investigation, Methodology, Project administration, Software, Supervision, Validation, Visualization, Writing – original draft, Writing – review & editing. JC: Conceptualization, Investigation, Methodology, Writing – original draft. YY: Methodology, Resources, Writing – review & editing. SS: Supervision, Validation, Writing – review & editing. YH: Investigation, Methodology, Writing – review & editing. JL: Funding acquisition, Project administration, Supervision, Writing – review & editing.
