# Supplementary figures and images for: Retroductal dexamethasone administration promotes the recovery from obstructive and inflammatory salivary gland dysfunction
Source: Front Immunol. 2024 Jul 9;15:1418703. doi: 10.3389/fimmu.2024.1418703 (PMC11263033; doi:10.3389/fimmu.2024.1418703)

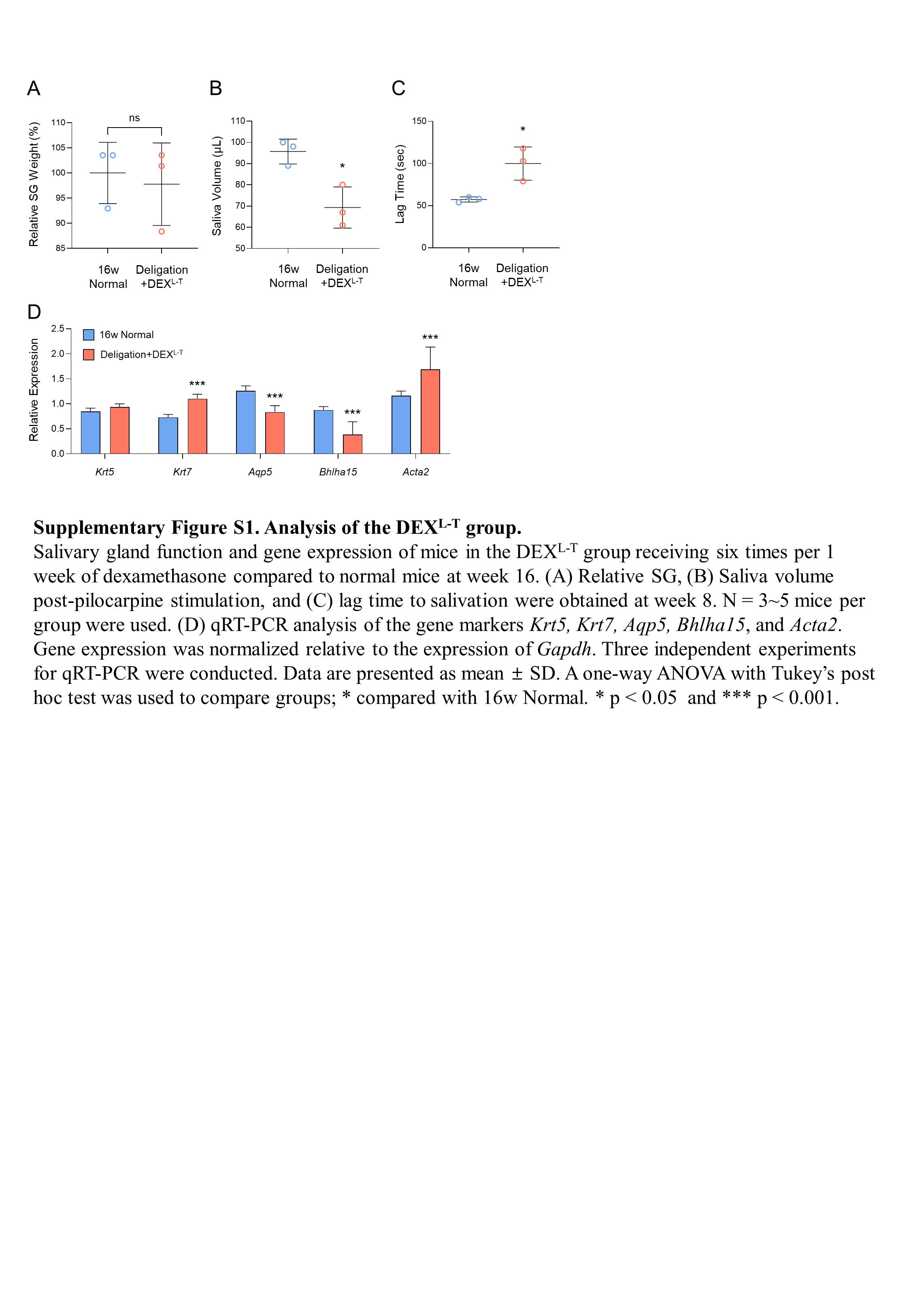

Supplement: Supplementary file 1 [file Image_1.jpeg]
