# Supplementary material for: Retroductal dexamethasone administration promotes the recovery from obstructive and inflammatory salivary gland dysfunction
Source: Front Immunol. 2024 Jul 9;15:1418703. doi: 10.3389/fimmu.2024.1418703 (PMC11263033; doi:10.3389/fimmu.2024.1418703)
Supplement: Supplementary file 2 [file Table_1.docx]

**Retroductal dexamethasone administation promotes the recovery from obstructive and inflammatory salivary gland dysfunction**

**Seungyeon Hwang**^1^**, Jae-Min Cho**^1^**, Yeo-Jun Yoon**^1^**, Sunyoung Seo**^1^**, Yong-Pyo Hong**^1^**, Jae-Yol Lim**^1^**^*^**

^1^ Department of Otorhinolaryngology, Yonsei University College of Medicine, Seoul, Republic of Korea

*** Correspondence:**Jae-Yol Lim, MD, PhD

Department of Otorhinolaryngology

Gangnam Severance Hospital

Yonsei University College of Medicine

211 Eonju-ro, Gangnam-gu, Seoul 06273, Republic of Korea

E-mail: jylimmd@yuhs.ac

Tel: 82-2-2019-3460

Fax: 82-2-3463-4750

**Supplementary Table 1.**

| **Supplementary Table 1. Primer sequence.** | | | |
| --- | --- | --- | --- |
| Species | Gene |  | Sequence |
| Mouse | *Gapdh* | Forward | CAT CAC TGC CAC CCA GAA GAC TG |
|  |  | Reverse | ATG CCA GTG AGC TTC CCG TTC AG |
| Mouse | *Krt5* | Forward | TTG GTG TTG GCA GTG GCT TT |
|  |  | Reverse | CCC GCT ACC CAA ACC AAG AC |
| Mouse | *Krt7* | Forward | GCT CTC GCT CCA CTG CTT AC |
|  |  | Reverse | CGC CAG CAA GCT CTG ATT GA |
| Mouse | *Aqp5* | Forward | ACG CTC CAC TAC TGA CAG GTC A |
|  |  | Reverse | GAA GTA GAG GAT TGC AGC CAG G |
| Mouse | *Bhlha15* | Forward | GCT GAC CGC CAC CAT ACT TAC |
|  |  | Reverse | TGT GTA GAG TAG CGT TGC AGG |
| Mouse | *Smgc* | Forward | TGG CTC TGC AAC ACA ACA GT |
|  |  | Reverse | GGC CAA AAG CTC CCA GGT AA |
| Mouse | *Bpifa2* | Forward | TGA ACA CAG CGG ACC TTG GCA A |
|  |  | Reverse | CCA TTG CCG TTG GAA GAC AGC T |
| Mouse | *Acta2* | Forward | GCC ATC ATG CGT CTG GAC TT |
|  |  | Reverse | ATC TCA CGC TCG GCA GTA GT |
| Mouse | *Tnf* | Forward | GGT GCC TAT GTC TCA GCC TCT T |
|  |  | Reverse | GCC ATA GAA CTG ATG AGA GGG AG |
| Mouse | *Il6* | Forward | TAC CAC TTC ACA AGT CGG AGG C |
|  |  | Reverse | CTG CAA GTG CAT CGT TGT TC |
| Mouse | *Il1b* | Forward | TGG ACC TTC CAG GAT GAG GAC A |
|  |  | Reverse | GTT CAT CTC GGA GCC TGT AGT G |
| Mouse | *Cd86* | Forward | ACG TAT TGG AAG GAG ATT ACA GCT |
|  |  | Reverse | TCT GTC AGC GTT ACT ATC CCG C |
| Mouse | *Ym1* | Forward | TAC TCA CTT CCA CAG GAG CAG G |
|  |  | Reverse | CTC CAG TGT AGC CAT CCT TAG G |
| Mouse | *Il10* | Forward | CGG GAA GAC AAT AAC TGC ACC C |
|  |  | Reverse | CGG TTA GCA GTA TGT TGT CCA GC |
| Mouse | *Cd163* | Forward | GGC TAG ACG AAG TCA TCT GCA C |
|  |  | Reverse | CTT CGT TGG TCA GCC TCA GAG A |
| Mouse | *Klf4* | Forward | CTA TGC AGG CTG TGG CAA AAC C |
|  |  | Reverse | TTG CGG TAG TGC CTG GTC AGT T |
| Human | *GAPDH* | Forward | GTC TCC TCT GAC TTC AAC AGC G |
|  |  | Reverse | ACC ACC CTG TTG CTG TAG CCA A |
| Human | *KRT5* | Forward | GCT GCC TAC ATG AAC AAG GTG G |
|  |  | Reverse | ATG GAG AGG ACC ACT GAG GTG T |
| Human | *KRT7* | Forward | TGT GGA TGC TGC CTA CAT GAG C |
|  |  | Reverse | AGC ACC ACA GAT GTG TCG GAG A |
| Human | *AQP5* | Forward | TAC GGT GTG GCA CCG CTC AAT G |
|  |  | Reverse | AGT CAG TGG AGG CGA AGA TGC A |
| Human | *STATH* | Forward | TTT TGC GTA GAA TTG GAA GAT TCG G |
|  |  | Reverse | AAT CAT GTC CTG CAG TTA CTG ATG |
| Human | *ACTA2* | Forward | CTA TGC CTC TGG ACG CAC AAC T |
|  |  | Reverse | CAG ATC CAG ACG CAT GAT GGC A |
| Human | *TNF* | Forward | CTC TTC TGC CTG CTG CAC TTT G |
|  |  | Reverse | ATG GGC TAC AGG CTT GTC ACT C |
| Human | *IL6* | Forward | AGA CAG CCA CTC ACC TCT TCA G |
|  |  | Reverse | TTC TGC CAG TGC CTC TTT GCT G |
| Human | *CCL2* | Forward | AGA ATC ACC AGC AGC AAG TGT CC |
|  |  | Reverse | TCC TGA ACC CAC TTC TGC TTG G |
| Human | *CXCL5* | Forward | CAG ACC ACG CAA GGA GTT CAT C |
|  |  | Reverse | TTC CTT CCC GTT CTT CAG GGA G |
| Human | *CXCL12* | Forward | CTC AAC ACT CCA AAC TGT GCC C |
|  |  | Reverse | CTC CAG GTA CTC CTG AAT CCA C |

**Supplementary Table 2.**

| **Supplementary Table 2. IF antibodies.** | | | |
| --- | --- | --- | --- |
| **Primary Antibodies** | | | |
| **Antibody** | **Company** | **Cat no.** | **Titration** |
| KRT5 | Biolegend | 905904 | 1:1000 |
| KRT7 | Abcam | 181598 | 1:1000 |
| AQP5 | Alomone | AQP-005 | 1:500 |
| BHLHA15 | Abcam | 187978 | 1:200 |
| KRT14 | Biolegend | 906004 | 1:400 |
| ACTA2 | Abcam | 124964 | 1:1000 |
| PECAM1 | Abcam | ab28364 | 1:50 |
| EPCAM | Abcam | ab71916 | 1:200 |
| TUBB3 | Cell signaling | 4466 | 1:100 |
| CDH1 | Cell signaling | 3195 | 1:300 |
| PDGFRB | Cell signaling | 3169 | 1:200 |
| **Secondary Antibodies** | | | |
| **Antibody** | **Company** | **Cat no.** | **Titration** |
| goat anti-chicken IgY, Alexa Fluor™ Plus 488 | Invitrogen | A32931 | 1:500 |
| goat anti-rabbit IgG, Alexa Fluor™ Plus 594 | Invitrogen | A32740 | 1:500 |
| goat anti-rabbit, Alexa Fluor™ Plus 488 | Invitrogen | A32731 | 1:500 |
| goat anti-mouse IgG, Alexa Fluor™ Plus 594 | Invitrogen | A32742 | 1:500 |
| goat anti-mouse IgG, Alexa Fluor™ Plus 488 | Invitrogen | A32723 | 1:500 |
| goat anti-rabbit IgG, Alexa Fluor™ Plus 594 | Invitrogen | A32740 | 1:500 |
